# “Going Flat” in Nipple‐Sparing Mastectomy—An Analysis of Patient‐Reported Outcome Measurements of Preliminary 57 Procedures

**DOI:** 10.1155/tbj/5032690

**Published:** 2026-03-27

**Authors:** Jun Su, Yi-Yuan Lee, Wen-Bin Kao, Nurul Hidayah Abdul Rauf, Bing-Fang Hwang, Chiu-Ying Chen, Mee-Hoong See, Hung-Wen Lai

**Affiliations:** ^1^ Department of General Surgery, Tan Tock Seng Hospital, Singapore, ttsh.com.sg; ^2^ Department of Public Health, China Medical University, Taichung, Taiwan, cmu.edu.cn; ^3^ Endoscopic & Oncoplastic Breast Surgery Center, Changhua Christian Hospital, Changhua, Taiwan, cch.org.tw; ^4^ Minimal Invasive Surgery Research Center, Changhua Christian Hospital, Changhua, Taiwan, cch.org.tw; ^5^ Division of Plastic and Reconstructive Surgery, Changhua Christian Hospital, Changhua, Taiwan, cch.org.tw; ^6^ Division of Breast and Endocrine Surgery, Selayang Hospital, Selayang, Malaysia; ^7^ Department of Occupational Safety and Health, China Medical University, Taichung, Taiwan, cmu.edu.tw; ^8^ Department of Surgery, Faculty of Medicine, Breast Surgery Unit, Universiti Malaya, Kuala Lumpur, Malaysia, um.edu.my; ^9^ Faculty of Medicine, Universiti Malaya, Kuala Lumpur, Malaysia, um.edu.my; ^10^ Department of Surgery, Division of Breast Surgery, Changhua Christian Hospital, Changhua, Taiwan, cch.org.tw; ^11^ School of Medicine, Chung Shan Medical University, Taichung, Taiwan, csmu.edu.tw

**Keywords:** breast cancer, going flat, immediate breast reconstruction, nipple-sparing mastectomy (NSM), patient-reported outcome

## Abstract

**Background:**

Nipple‐sparing mastectomy (NSM) with immediate breast reconstruction (IBR) has become an increasingly popular procedure offering satisfactory aesthetic outcomes with adequate oncologic safety. However, outcomes of NSM without IBR have been rarely evaluated. We aim to assess the clinical outcomes of NSM in patients refusing IBR with a focus on patient‐reported outcome measurements.

**Methods:**

This retrospective study enrolled female breast cancer patients who underwent NSM but refused IBR from February 2010 to April 2022 in a single institution. Clinical outcomes and patient‐reported aesthetic results were evaluated and analyzed.

**Results:**

During the study period, there were 57 cases of primary breast cancer patients who underwent NSM but refused IBR. The mean operative duration was 186.6 min, and median length of hospital stay was 4 days. Median intraoperative blood loss was 30 mL with the median resected specimen weight at 360.5 g. There was no incidence of margin involvement in this study. The complication rate was 12.3% with all of them being minor complications with Clavien–Dindo I and II scores. Locoregional recurrence and distant metastasis during the study period were 5.3% and 1.8%, respectively, with a mean follow‐up period of 64.4 months. There was no mortality during the study duration. Aesthetic outcomes assessed via the BREAST‐Q questionnaire were satisfactory with mean scores ranging between 35.1 and 68.4.

**Conclusion:**

This study concludes that NSM in patients refusing IBR is an alternative compared to conventional mastectomy with acceptable aesthetic outcomes. These findings underscore the importance of individualized patient choices in breast cancer treatment.

**SYNOPSIS**
 Our current retrospective study of using patient‐reported outcome measurements to assess aesthetic outcomes of nipple‐sparing mastectomy (NSM) in patients refusing immediate breast reconstruction (IBR) shows that postoperative patient satisfaction was adequate with BREAST‐Q questionnaire mean values ranging from 35.1 to 68.4. Locoregional recurrence and distant metastasis were 5.3% and 1.8%, respectively, with a mean follow‐up period of 64.4 months. No mortality was reported during the study period.

## 1. Introduction

Breast cancer remains the most prevalent cancer worldwide for females, with more than 2 million newly diagnosed cases in 2020 [1, 2]. Despite extensive advances in breast cancer research, total mastectomy or breast‐conserving surgery with radiation for suitable candidates remains the mainstay of surgical treatment. Total mastectomy, which involves the removal of the nipple–areolar complex (NAC) and skin envelope and creation of a transverse chest wall scar, is associated with low long‐term locoregional recurrence rates of 2%–3% [3]. This however sometimes leaves a cosmetic deformity with psychological sequelae which are difficult to treat [4].

Nipple‐sparing mastectomy (NSM) was originally developed by Freeman in the 1960s when immediate breast reconstruction (IBR) techniques became available and was touted as a procedure to remove the entire mammary gland while preserving the overlying skin and NAC. With resurgence in bilateral prophylactic mastectomy in the setting of high‐risk breast cancer syndromes such as BRCA mutations, there has been renewed interest in NSM as an oncologically safe and yet aesthetically satisfactory procedure for breast cancer [5–7]. Current evidence has shown that NSM is oncologically safe and is not associated with higher locoregional recurrence compared to total mastectomy [8–11]. As such, NSM with IBR has become an important technique in the armamentarium of many breast surgeons.

However, some patients in our experience have opted for the combined option of NSM in preserving the skin flap and NAC but refusing IBR. Mastectomy without IBR or colloquially known as “going flat” has been prominent in the public domain since 2011 with further prominence in 2016 driven by The New York Times article entitled, “Going Flat After Breast Cancer” [12, 13]. Reasons often quoted for refusing IBR range from not wanting to undergo more extensive surgery, concerns about interference with cancer treatment or detection of cancer recurrence, fears about IBR complications, concerns regarding the safety of breast implants, and the lack of financial resources or insurance [14, 15]. We hypothesized that NSM without IBR could still represent an aesthetically acceptable outcome in patients who have concerns about IBR risks. In this study, we retrospectively reviewed women who had undergone NSM without reconstruction in our database and reviewed their satisfaction regarding this procedure via patient‐reported outcome measurements (PROMs). To provide better understanding on the effect of IBR on patient satisfaction, we also compared PROM results between patients who had NSM with and without IBR during the same time period.

Until now, “going flat” combined with the NSM procedure has been unexplored in breast cancer literature, and we hope to provide a better understanding of this unique procedure.

## 2. Materials and Methods

### 2.1. Study Design and Patient Recruitment

We reviewed records from our prospectively collected database and identified female breast cancer patients who underwent NSM but refused IBR. Demographic data, clinical and pathological data, treatment regimens and outcomes, and survival were obtained from the electronic medical records of patients treated at Changhua Christian Hospital (CCH), a tertiary hospital in Central Taiwan, from 1 February 2010 to 30 April 2022.

Women were included if they had presented with primary operable disease and had undergone NSM without IBR. They would be included regardless of surgical approach (minimal access [endoscopic‐ or robotic‐assisted] or conventional NSM) so long as all relevant clinicopathological data regarding the initial disease presentation and treatment were available. Patients who did not undergo mastectomy or had insufficient information were excluded. Additionally, patients who did not receive patient‐reported outcome survey or did not respond to the questionnaire were also excluded. Separately, we also included patients who had undergone NSM with IBR during the same time period as a control group to compare PROM results between the two groups.

**FIGURE 1:**
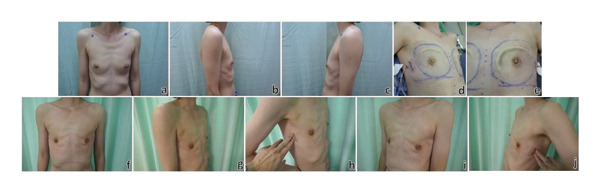
Clinical photos of nipple‐sparing mastectomy (NSM) without reconstruction. (a–c) Preoperative images of a 44‐year‐old female with left breast ductal carcinoma in situ who underwent left therapeutic NSM and right prophylactic NSM without reconstruction. (d, e) Intraoperative photos after completion of bilateral NSM. (f–j) Postoperative photos 1 week after surgery.

The clinical outcomes of all patients were evaluated, incorporating parameters such as operative time, intraoperative blood loss, surgical specimen weight, margin status, length of hospital stay, postoperative complications, local recurrence rate, and overall survival. Additionally, aesthetic outcomes regarding cosmetic appearance and quality of life (QoL) were assessed using patient‐reported questionnaires. The BREAST‐Q 2.0 Mastectomy module questionnaire and a 13‐question survey developed in our institution for PROMs were used. 4 of the Mastectomy module questionnaire scales were used, namely, the “satisfaction with breasts,” “psychosocial well‐being,” “sexual well‐being,” and “physical well‐being: chest” scales [16]. Summated scores were then converted to the equivalent Rasch‐transformed score which has a range of 0–100 with higher scores representing a better outcome. Follow‐up data were collected until 30 June 2024. Clinical photos demonstrating the aesthetic result of NSM without IBR are illustrated in Figure 1. All patients have given consent for the publication of their photos.

The primary outcomes of this study were surgical complications, local recurrence, and overall survival. Surgical complications, including infection rate, seroma formation, hematoma, skin flap and nipple ischemia or necrosis, and wound healing problems, were assessed and graded for severity using the Clavien–Dindo (CD) score [17]. PROMs were also evaluated, focusing on satisfaction with cosmetic appearance, QoL, and patient preferences for reconstruction, nipple preservation, and minimal access (endoscopic‐ or robotic‐assisted) surgical approaches.

This study has been ethically approved by the Institutional Review Board of the CCH (CCH IRB No. 231217).

### 2.2. Statistical Analysis

Patient characteristics and clinical variables were analyzed using descriptive statistical analysis. Categorical variables were reported as frequency and percentage. Continuous variables were presented as mean ± standard deviation (SD) or median with interquartile range (IQR), based on the normality of distribution assessed by the Shapiro–Wilk test. Comparisons between groups were performed using the independent two‐sample *t*‐test or the Mann–Whitney *U* test, as appropriate. Categorical variables were compared using the chi‐square test or Fisher’s exact test. Survival outcomes were estimated using the Kaplan–Meier method. All statistical analyses were conducted using R software (Version 4.5.0; R Foundation for Statistical Computing, Vienna, Austria). All statistical tests were two‐sided, and a *p* value < 0.05 was considered statistically significant.

## 3. Results

### 3.1. Clinicopathological Characteristics

During the study period, there were 57 cases of breast cancer patients who had primary operable disease and underwent NSM but refused breast reconstruction. All these patientsresponded to PROMs questionnaire and were enrolled. The median age of patients was 55 years old, and BMI was 24.8 kg/m^2^. The primary indication for surgery was invasive ductal carcinoma, representing 61.8% followed by ductal carcinoma in situ, which accounted for 20% of the study population. The majority (83.7%) of patients had early‐stage breast cancer from Stage 0 to 2 with luminal A immunohistochemical subtype being the most common (31.9%). The baseline patient characteristics and tumor biology are presented in Table 1.

**TABLE 1:** Baseline demographics and clinicopathologic characteristics of patients who underwent nipple‐sparing mastectomy without breast reconstruction.

	Total *N* = 57 (%)
Median age, years [IQR]	55 [48, 63]
Below 40	2 (3.5)
40–59	33 (57.9)
60 and above	22 (38.6)
Median body mass index (kg/m^2^) [IQR]	24.8 [22.2, 26.7]
< 18	4 (7)
18–24	21 (36.8)
≧ 24	32 (56.1)

*Laterality*	
Right	25 (43.9)
Left	32 (56.1)

*Postoperation tumor histology (NA = 2)*	
Benign^∗^	1 (1.8)
pCR	5 (9.1)
DCIS	11 (20)
IDC	34 (61.8)
ILC	2 (3.6)
Mucinous carcinoma	2 (3.6)

*Type of nipple-sparing mastectomy*	
Conventional	19 (33.3)
Endoscopic	37 (64.9)
Robotic	1 (1.8)
Axillary surgery	
SLNB	42 (73.7)
SLNB then ALND	6 (10.5)
ALND	8 (14)
Not performed	1 (1.8)
Median pathological tumor size, cm [IQR]	2.2 [1, 2.6]

*Lymph node metastasis (NA = 2)*	
Yes	14 (25.5)
No	41 (74.5)

*Lymph node stage*	
N0	43 (75.4)
N1	11 (19.3)
N2	3 (5.3)

*Pathological stage (NA = 2)*	
0	11 (20)
pCR	5 (9.1)
I	15 (27.3)
II	20 (36.4)
III	4 (7.3)

*Grade (NA = 7)*	
I	6 (12)
II	33 (66)
III	11 (22)
*ER (NA = 5)*	
Positive	44 (84.6)
Negative	8 (15.4)
PR (NA = 5)	
Positive	37 (71.2)
Negative	15 (28.8)

*HER-2 (NA = 11)*	
Positive	14 (30.4)
Negative	32 (69.6)

*Ki-67 (NA = 19)*	
≦ 20	25 (65.8)
> 20	13 (34.2)

*Immunohistochemical subtype (NA = 10)*	
Luminal A	15 (31.9)
Luminal B1	14 (29.8)
Luminal B2	10 (21.3)
HER‐2	5 (10.6)
TNBC	3 (6.4)

*Endocrine therapy*	
Yes	42 (73.7)
No	15 (26.3)

*Chemotherapy*	
Yes	35 (61.4)
No	22 (38.6)

*Radiotherapy*	
Yes	23 (40.4)
No	34 (59.6)

*Neoadjuvant chemotherapy*	
Yes	19 (33.3)
No	38 (66.7)

*Note:* NA: no record available; HER‐2: human epidermal growth factor receptor 2.

Abbreviations: ALND, axillary lymph node dissection; DCIS, ductal carcinoma in situ; ER, estrogen receptor; IDC, infiltrating ductal carcinoma; ILC, infiltrating lobular carcinoma; IQR, interquartile range; pCR, pathological complete response; PR, progesterone receptor; SD, standard deviation; SLNB, sentinel lymph node biopsy; and TNBC, triple‐negative breast cancer.

^∗^Performed for indication of DCIS on excision biopsy; however, there was no residual malignancy in mastectomy specimen.

Additionally, for PROM comparative analysis, there were 264 patients who underwent NSM with breast reconstruction and completed PROM questionnaire during the study period. The responses given by this group were compared and analyzed with the PROM data from the NSM without reconstruction group (Table 2).

**TABLE 2:** Patient‐reported outcome measurements comparing nipple‐sparing mastectomy with and without reconstruction.

BREAST‐Q version 2.0			
Scale	NSM without reconstruction (*N* = 57)	NSM with reconstruction (*N* = 264)	*p* value
Satisfaction with breasts	50.4 ± 13.2	59.4 ± 14.4	< 0.001
Psychosocial well‐being	55.5 ± 15.0	65.2 ± 16.7	< 0.001
Sexual well‐being	35.1 ± 21.1	48.5 ± 18.7	< 0.001
Physical well‐being: chest	68.7 ± 20.9	34.8 ± 16.4	< 0.001

**Mean score for questionnaire surveying aesthetic outcomes and satisfaction in our institute**			
**Questions**	**NSM without reconstruction (*N* = 57)**	**NSM with reconstruction (*N* = 264)**	***p* value**

[Q1] Postoperative breast appearance satisfaction—with clothes	2.3 ± 0.7	3.1 ± 0.8	< 0.001
[Q2] Postoperative breast appearance satisfaction—without clothes	2.1 ± 0.9	2.7 ± 0.9	< 0.001
[Q3] Postoperative bilateral breast size satisfaction	2.2 ± 0.8	2.7 ± 0.9	< 0.001
[Q4] Postoperative bilateral breast symmetry satisfaction	2.3 ± 1.0	2.7 ± 0.9	0.01
[Q5] Postoperative nipple–areola position satisfaction	2.6 ± 0.9	3.0 ± 0.8	0.01
[Q6] Scar appearance satisfaction	2.8 ± 0.9	2.9 ± 0.9	0.18
[Q7] Scar length satisfaction	2.7 ± 0.9	3.0 ± 0.9	0.03
[Q8] Surgical wound position satisfaction	2.8 ± 0.8	3.1 ± 0.9	0.04

### 3.2. Operative Procedure and Oncologic Outcomes

Among these 57 NSM without breast reconstruction procedures, 64.9% were performed via endoscopic approach followed by 33% conventional and 1.8% robotic‐assisted. Sentinel lymph node biopsy was the most commonly performed axillary procedure (73.7%) with only 14% of patients receiving upfront axillary lymph node dissection. The mean operative duration was 186.6 ± 70 min, and median length of hospital stay was 4 days. Median intraoperative blood loss was 30 mL with the median resected specimen weight at 360.5 g. There was no incidence of margin involvement in this study (Table 3).

**TABLE 3:** Clinical outcomes of patients who underwent nipple‐sparing mastectomy without reconstruction.

Clinical outcome	Total (*N* = 57)
Mean surgery duration, min ± SD (range)	186.6 ± 70 (54–385)
Median blood loss, ml [IQR]	30 [20, 40]
Median specimen weight, grams [IQR]	360.5 [309, 468.8]
Median duration of hospitalization, days [IQR]	4 [3, 4]
Complications (%)	
Yes	7 (12.3)
No	50 (87.7)
Complication type (%)	
Delayed wound healing	0 (0)
Skin blister from thermal injury	0 (0)
Seroma requiring frequent aspiration > 6 weeks	3 (5.3)
Hematoma	1 (1.8)
Wound infection	3 (5.3)
NAC ischemic necrosis	0 (0)
Small partial skin flap ischemia/necrosis	1 (1.8)
Clavien–Dindo score	
0	50 (87.7)
I	5 (8.8)
II	2 (3.5)
III‐V	0 (0)
Follow‐up, months ± SD	64.4 ± 35.9
Margin involved (NA = 1)	
Yes	0 (0)
No	56 (100)
Recurrence (%)	
Yes	3 (5.3)
No	54 (94.7)
Metastasis (%)	
Yes	1 (1.8)
No	56 (98.2)
Mortality (%)	
Yes	0 (0)
No	57 (100)

*Note:* NA: no record available; IQR: interquartile range.

Abbreviations: NAC, nipple–areolar complex; SD, standard deviation.

In terms of postoperative outcomes, 3 (5.3%) patients had minor wound infection which was treated with outpatient oral antibiotics while another 3 (5.3%) patients had postoperative seroma which required frequent outpatient aspirations of more than 6‐week duration. There was also 1 case of postoperative hematoma and 1 case of partial skin flap necrosis which were treated nonoperatively and resolved successfully. The median follow‐up time of the patients was 64.4 ± 35.9 months. Locoregional recurrence and distant metastasis during the study period were 5.3% and 1.8%, respectively. There were no cases of mortality during the study duration. Kaplan–Meier curves illustrating the oncologic outcomes in terms of overall survival, disease‐free survival, recurrence‐free survival, and metastasis‐free survival are depicted in Figure 2. The clinical results and oncologic outcomes are summarized in Table 3.

FIGURE 2Kaplan–Meier survival curves of patients who underwent nipple‐sparing mastectomy without reconstruction. (a) Overall survival. (b) Disease‐free survival. (c) Recurrence‐free survival. (d) Metastasis‐free survival.

**Figure:**
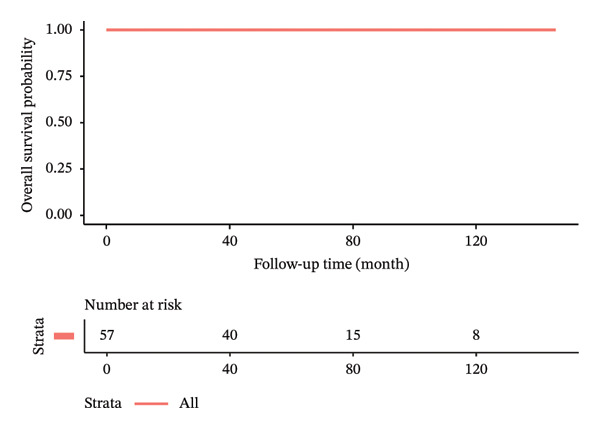
(a)

**Figure:**
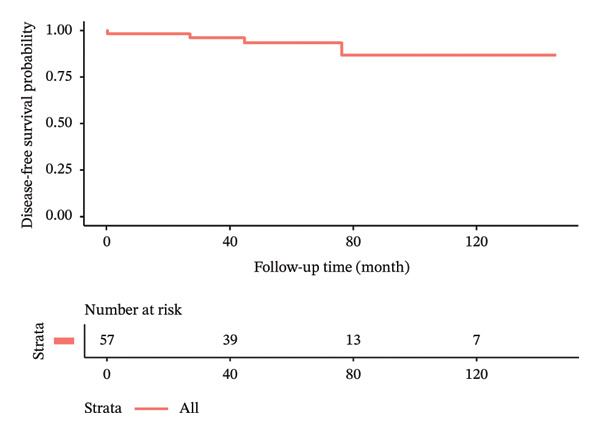
(b)

**Figure:**
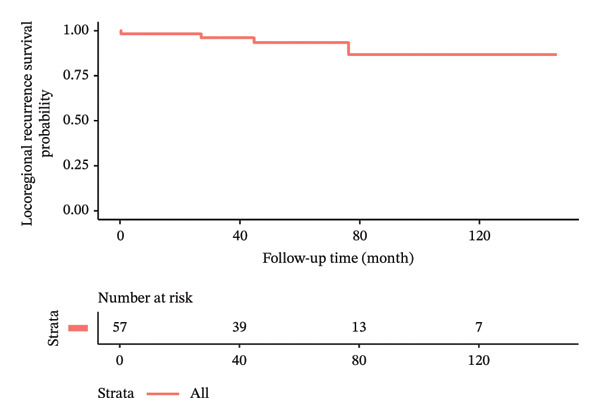
(c)

**Figure:**
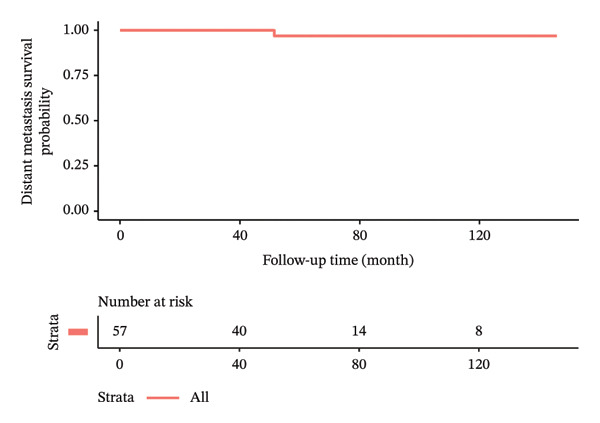
(d)

### 3.3. Patient‐Reported Outcome

In this study, the postoperative aesthetic outcome of patients who underwent NSM but refused IBR (Figure 1) was surveyed by using both the BREAST‐Q 2.0 Mastectomy module questionnaire as well as a 13‐question survey to evaluate patient‐reported outcomes. In the satisfaction with breasts scale, the mean score was 50.4 ± 13.2 with the majority of patients being satisfied with their postoperative clothed look and the fitting of their clothing postoperatively (73.7% and 78.5%, respectively).

In the psychosocial well‐being scale, the mean score was 55.5 ± 15.0. Low scores were noted in the social confidence, femininity, and attractiveness questions with only 38.6%, 29.8%, and 24.5% of patients being satisfied in these areas most or all of the time. Higher scores were given in the emotional health, equal worth, and body acceptance questions with 56.2%, 57.9%, and 61.5% of patients being satisfied in respective areas.

In the sexual well‐being scale, the mean score was significantly lower at 35.1 ± 21.1. The number of patients having satisfaction in each area most or all of the time ranged from 15.8% to 21.1%. The mean score was significantly higher at 68.4 ± 21.6 for the physical well‐being: chest scale. Postoperative pain and discomfort were low in this study with 70.2% of patients having no aching sensation in the breast area and 54.4% of patients having no difficulty lifting or moving their arms.

Using the institutional questionnaire evaluating postoperative aesthetic outcomes and satisfaction, patients reported mostly “Good” and “Excellent” responses to questions relating to their impression of postoperative scar length and breast appearance. The mean score ranged from 2.1 to 2.8 (out of maximum score of 4). In terms of whether patients would opt for NSM without breast reconstruction if they had to choose again, 84.2% of patients chose “Yes.” All patients responded “Yes” when asked if they would want NAC preservation if they had to choose again. 53 (93%) of respondents would opt not for breast reconstruction if they had to choose again. Finally, 52 (91.2%) of patients would choose for minimal access (endoscopic‐assisted or robotic‐assisted) NSM if they had to choose again. The results for patient‐reported outcomes are summarized in Table 4.

**TABLE 4:** Patient‐reported outcome measurements after nipple‐sparing mastectomy without reconstruction.

BREAST‐Q Version 2.0						
**Satisfaction with breasts**						
**With your breast area in mind, in the past week, how satisfied or dissatisfied have you been with:**	**Very dissatisfied**	**Somewhat dissatisfied**		**Somewhat satisfied**	**Very satisfied**	**Mean ± SD**
a. How you look in the mirror clothed?	2 (3.5)	13 (22.8)		41 (71.9)	1 (1.8)	50.4 ± 13.2
b. How comfortably your bras fit?	1 (1.8)	11 (19.6)		40 (71.4)	4 (7.1)	
c. Being able to wear clothing that is more fitted?	3 (5.5)	24 (43.6)		26 (47.3)	2 (3.6)	
d. How you look in the mirror unclothed?	5 (8.8)	24 (42.1)		25 (43.9)	3 (5.3)	

**Psychosocial well-being**						
**With your breast area in mind, in the past week, how often have you felt:**	**None of the time**	**A little of the time**	**Some of the time**	**Most of the time**	**All of the time**	**Mean ± SD**

a. Confident in a social setting?	1 (1.8)	7 (12.3)	27 (47.4)	17 (29.8)	5 (8.8)	55.5 ± 15.0
b. Emotionally able to do the things that you want to do?	1 (1.8)	10 (17.5)	20 (35.1)	20 (35.1)	6 (10.5)	
c. Emotionally healthy?	1 (1.8)	4 (7.0)	20 (35.1)	25 (43.9)	7 (12.3)	
d. Of equal worth to other women?	2 (3.5)	6 (10.5)	16 (28.1)	24 (42.1)	9 (15.8)	
e. Self‐confident?	2 (3.5)	4 (7.0)	23 (40.4)	20 (35.1)	8 (14.0)	
f. Feminine in your clothes?	4 (7.0)	15 (26.3)	21 (36.8)	13 (22.8)	4 (7.0)	
g. Accepting of your body?	1 (1.8)	3 (5.3)	18 (31.6)	23 (40.4)	12 (21.1)	
h. Normal?	1 (1.8)	8 (14.0)	13 (22.8)	23 (40.4)	12 (21.1)	
i. Like other women?	3 (5.3)	10 (17.5)	16 (28.1)	18 (31.6)	10 (17.5)	
j. Attractive?	5 (8.8)	18 (31.6)	20 (35.1)	10 (17.5)	4 (7.0)	

**Sexual well-being**						
**Thinking of your sexuality, how often do you generally feel:**	**None of the time**	**A little of the time**	**Some of the time**	**Most of the time**	**All of the time**	**Mean ± SD**

a. Sexually attractive in your clothes?	12 (21.1)	20 (35.1)	13 (22.8)	9 (15.8)	3 (5.3)	35.1 ± 21.1
b. Comfortable/at ease during sexual activity?	22 (38.6)	14 (24.6)	11 (19.3)	6 (10.5)	4 (7.0)	
c. Confident sexually?	23 (40.4)	12 (21.1)	12 (21.1)	7 (12.3)	3 (5.3)	
d. Satisfied with your sex life?	24 (42.1)	11 (19.3)	11 (19.3)	8 (14.0)	3 (5.3)	
e. Confident sexually about how your breast area looks when unclothed?	21 (36.8)	17 (29.8)	10 (17.5)	7 (12.3)	2 (3.5)	
f. Sexually attractive when unclothed?	20 (35.1)	15 (26.3)	13 (22.8)	6 (10.5)	3 (5.3)	

**Physical well-being: chest**						
**In the past week, how often have you experienced:**	**None of the time**	**Some of the time**		**All of the time**		**Mean ± SD**

a. Pain in the muscles of your chest?	23 (40.4)	30 (52.6)		4 (7.0)		68.4 ± 21.6
b. Difficulty lifting or moving your arms?	31 (54.4)	23 (40.4)		3 (5.3)		
c. Difficulty sleeping because of discomfort in your breast area?	32 (56.1)	19 (33.3)		6 (10.5)		
d. Tightness in your breast area?	22 (38.6)	30 (52.6)		5 (8.8)		
e. Pulling in your breast area?	24 (42.1)	28 (49.1)		5 (8.8)		
f. Nagging feeling in your breast area?	34 (59.6)	20 (35.1)		3 (5.3)		
g. Tenderness in your breast area?	18 (31.6)	23 (40.4)		16 (28.1)		
h. Sharp pains in your breast area?	33 (57.9)	23 (40.4)		1 (1.8)		
i. Aching feeling in your breast area?	40 (70.2)	14 (24.6)		3 (5.3)		
j. Throbbing feeling in your breast area?	27 (47.4)	27 (47.4)		3 (5.3)		

A questionnaire surveying aesthetic outcomes and satisfaction in our institute**^∗^**						
**Questions (*N* = 57), *N* (%)**	**Poor**	**Fair**		**Good**	**Excellent**	**Mean ± SD**

[Q1] Preoperative breast appearance satisfaction	3 (5.3)	26 (45.6)		21 (36.8)	7 (12.3)	2.6 ± 0.8
[Q2] Postoperative breast appearance satisfaction—with clothes	3 (5.3)	36 (63.2)		14 (24.6)	4 (7.0)	2.3 ± 0.7
[Q3] Postoperative breast appearance satisfaction—without clothes	14 (25.0)	26 (46.4)		12 (21.4)	4 (7.1)	2.1 ± 0.9
[Q4] Postoperative bilateral breast size satisfaction	8 (18.6)	23 (53.5)		9 (20.9)	3 (7.0)	2.2 ± 0.8
[Q5] Postoperative bilateral breast symmetry satisfaction	9 (22.5)	17 (42.5)		9 (22.5)	5 (12.5)	2.3 ± 1.0
[Q6] Postoperative nipple–areola position satisfaction	4 (7.0)	26 (45.6)		16 (28.1)	11 (19.3)	2.6 ± 0.9
[Q7] Scar appearance satisfaction	5 (8.8)	18 (31.6)		20 (35.1)	14 (24.6)	2.8 ± 0.9
[Q8] Scar length satisfaction	5 (8.8)	19 (33.3)		22 (38.6)	11 (19.3)	2.7 ± 0.9
[Q9] Surgical wound position satisfaction	2 (3.5)	19 (33.3)		22 (38.6)	14 (24.6)	2.8 ± 0.8
—	Yes	No		Not sure	—	—
[Q10] If you could choose again, would you choose to undergo nipple‐sparing mastectomy without reconstruction?	48 (84.2)	3 (5.3)		6 (10.5)	—	—
[Q11] Would you want to receive breast reconstruction if you had the chance to choose again?	4 (7)	53 (93)		—	—	—
[Q12] Would you want to preserve your nipple–areolar complex if you had the chance to choose again?	57 (100)	0 (0)		—	—	—
[Q13] Would you want to receive minimal access (endoscopic‐assisted and robotic‐assisted) NSM if you had the chance to choose again?	52 (91.2)	5 (8.8)		—	—	—

Abbreviation: NSM = nipple‐sparing mastectomy.

^∗^For Q1–Q9: Poor: 1 point, Fair: 2 points, Good: 3 points, and Excellent: 4 points.

Comparing results between the NSM with reconstruction and NSM without reconstruction groups, the reconstruction group had significantly higher BREAST‐Q mean scores in the satisfaction with breasts, psychosocial well‐being, and sexual well‐being (*p* < 0.001 for all 3 statistical analyses). The NSM with reconstruction group also scored significantly higher for all 8 questions in our institutional questionnaire. Conversely, the NSM without reconstruction group had higher mean score in the physical well‐being: chest scale (68.7 ± 20.9 vs. 34.8 ± 16.4, *p* < 0.001, Table 2).

## 4. Discussion

Preserving the NAC is important for maintaining the integrity of the female breast identity. For some women, the loss of the NAC is viewed as a form of female mutilation, underscoring the significance of safeguarding it whenever possible to retain the original appearance and aesthetics of the breast. This is well supported by existing literature showing that patients who underwent NSM had a higher sexual and psychosocial well‐being score in the BREAST‐Q domain compared to SSM [18]. Our paper concurs with this finding in that all patients would choose NAC preservation if they had to choose again, suggesting that in selected patient populations, NAC preservation may be aesthetically more important than breast reconstruction.

Our study showed that NSM without reconstruction is associated with acceptable postoperative and oncologic outcomes. Complication rates were low at 12.3% with all of these being CD score I and II complications which resolved with outpatient care. The majority of our patients were also older, with 57.9% of them being between 40 and 59 years of age and 38.6% of them being 60 years of age and more. These findings expectedly suggest that elderly patients with more comorbidities and surgical risk may prefer a shorter and less complicated procedure without breast reconstruction. These preferences were also noted by Morrow et al. who found that increased age and major comorbidity were associated with not undergoing reconstruction after mastectomy [15].

Importantly, our study demonstrates that patients who preferred NSM but refused reconstruction were satisfied in terms of the cosmetic outcomes. The institutional questionnaire results show that most patients were satisfied with their postoperative appearance and scar length. 91.2% of patients would also opt for minimal access NSM if they had to choose again. This is likely due to the fact that minimal access NSM allows for a smaller and more aesthetically placed scar well hidden in the axilla or lateral chest wall [19].

Johnson et al. showed that patients who declined post‐mastectomy reconstruction more frequently had a greater burden of local disease and required adjuvant chemotherapy or radiotherapy [13]. Indeed, another large study surveying the patient‐reported outcomes of 931 patients concluded that the top two reasons for “going flat” were wishes for a faster recovery and the avoidance of a foreign body [20]. Increasing fears regarding breast reconstruction complications, which can occur in up to 33% in some studies, likely contribute to this as well [21]. This was supported by our results where a significant number of patients (40.4% for radiotherapy and 61.4% for chemotherapy) required further adjuvant treatment.

Focusing our attention on the BREAST‐Q questionnaire results, we note that high scores were rated in the “satisfaction with breasts,” “psychosocial well‐being,” and “physical well‐being: chest” sections with mean scores ranging from 50.4 to 68.4. However, “sexual well‐being” was rated significantly lower at a mean score of 35.1. This is consistent with the findings of Casaubon and Cornell et al. showing that the diagnosis of breast cancer significantly impacts baseline sexual function, with NSM with reconstruction patients having less satisfaction than patients who underwent breast‐conserving surgery [22, 23]. Despite this, Tomita et al. showed that patients with simple mastectomy alone had much lower mean “sexual well‐being” score of 29.9 [24]. This suggests that the preservation of the NAC aids in the preservation of sexual well‐being and body image, although the benefits may be limited compared to patients who have had breast‐conserving surgery. These significant findings will aid patient counseling regarding the types of surgical options for breast cancer and the impact they may have on QoL on top of their oncological outcomes. Through this study, we have also demonstrated that NSM without reconstruction is an oncologically and clinically safe procedure with low complication rates and acceptable oncological outcomes (Table 3) [25].

Additionally, our comparative analysis of mean BREAST‐Q scores between patients who underwent NSM with and without reconstruction showed that these scores were significantly higher in the domains of satisfaction with breasts, psychosocial well‐being, and sexual well‐being for patients who had NSM with reconstruction. These patients who had NSM with reconstruction also scored higher on our institutional questionnaire. On the contrary, the patients who underwent NSM without reconstruction scored significantly higher on the physical well‐being: chest scale compared to the patients who underwent NSM with reconstruction. The physical well‐being: chest BREAST‐Q scale focuses predominantly on arm mobility as well as pain and discomfort in the chest area [16], and the lower scores in the reconstruction group could be related to increased postoperative pain and discomfort especially in autologous reconstruction [26]. This highlights a key aspect in breast reconstruction decision‐making and patient counseling in that NSM with reconstruction may provide better aesthetic outcomes than NSM without reconstruction, but this may come with a trade‐off of increased postoperative pain and reduced arm mobility.

We acknowledge that some of our aesthetic outcomes occur as a result of minimal access breast surgery such as endoscopic‐assisted and robotic‐assisted NSM, and these may not be generalizable to all healthcare institutions due to the need for specialized training and equipment. Secondly, cost considerations especially in the setting of robotic‐assisted NSM may preclude the widespread use of these surgical techniques. Lastly, it has been noted that insurance coverage and socioeconomic conditions are important considerations in a patient’s decision to pursue post‐mastectomy reconstruction [15]. Patients in the Taiwan healthcare system do not routinely receive reimbursement from the National Health Insurance for breast reconstruction procedures, and as such this could be a significant factor in the decision‐making process of our study population.

The main limitation of our study is its retrospective nature which may contribute to recall bias especially regarding qualitative aspects such as patient satisfaction and psychosocial well‐being regarding the surgery. Secondly, the median follow‐up of 64.4 months may not be adequate to detect late recurrences or distant metastases in our patient population. This is importantsince the majority of our patients (83%) have luminal subtype breast cancer which has an inclination toward late recurrences [27]. Lastly, despite the use of standardized questionnaires to evaluate postoperative patient satisfaction, the individual patient and surgeon shared decision‐making process leading to the final decision for NSM without reconstruction is difficult to capture in our study. Further qualitative studies involving structured interviews and open‐ended input from both patients and surgeons may provide additional insight into this area.

Our study provides an alternative view to NSM without IBR as an aesthetically viable option for patients who wish to proceed with “going flat” after mastectomy and can be part of a comprehensive discussion regarding surgical options in breast cancer. With increasing public awareness of the risks and benefits of post‐mastectomy breast reconstruction, there is a need to engage the patient in a thorough shared decision‐making process to arrive at a personalized treatment plan for each patient [28]. This will allow for better patient satisfaction regarding the final surgical option chosen and improved patient–surgeon communication [29].

As such, we believe this study provides key insights into the decision‐making process and outcomes of patients undergoing NSM with or without reconstruction. Specifically, the use of PROMs in our study will hopefully contribute to a growing body of literature on this unique procedure.

## 5. Conclusions

In conclusion, we have demonstrated that in patients who opted for NSM but refused breast reconstruction, NSM alone is an oncologically safe procedure with low complication rates and satisfactory aesthetic outcomes. NAC preservation is a key feature which may be highly valued by select patients over breast reconstruction. We recommend that the decision to perform NSM with or without reconstruction should be a personalized choice incorporating patient wishes, oncologic factors, and existing comorbidities and physiological state.

## Author Contributions

Jun Su and Hung‐Wen Lai were involved in manuscript drafting. Yi‐Yuan Lee was responsible for statistical analysis and interpretation of data. Hung‐Wen Lai and Nurul Hidayah Abdul Rauf were involved in manuscript editing. Nurul Hidayah Abdul Rauf, Mee‐Hoong See, and Wen‐Bin Kao were involved in study conceptualization. Chiu‐Ying Chen provided supervision and critical revision. Bing‐Fang Hwang provided supervision. All the authors met to review this manuscript.

## Funding

This research received funding from Changhua Christian Hospital, and the funding information is as follows: 111‐CCH‐IRP‐089, 114‐CCH‐IRP105, and 114‐CCH‐ICO‐153.

## Ethics Statement

This study was approved by the CCH IRB (CCH IRB No. 231217) and conformed to the Declaration of Helsinki 1975.

## Consent

All individuals have given their written informed consent for publication of their data in this study.

## Conflicts of Interest

The authors declare no conflicts of interest.

## Data Availability

The data that support the findings of this study are available from the corresponding authors upon reasonable request.
